# Estimation of discrete mixed Poisson-Erlang distribution with applications to medical data

**DOI:** 10.1371/journal.pone.0331472

**Published:** 2025-09-15

**Authors:** Mohamed Ahmed Mosilhy, Sadiah M. A. Aljeddani, Mahmoud H. Abu-Moussa

**Affiliations:** 1 Department of Mathematics, Faculty of Science, Cairo University, Giza, Egypt; 2 Mathematics Department, Al-Lith University College, Umm Al-Qura University, Al-Lith, Saudi Arabia; 3 Faculty of Education and Arts, Sohar University, Sohar, Oman; University of Malaya, MALAYSIA

## Abstract

This paper discusses the estimation of the discrete mixed Poisson-Erlang distribution (DMPED). Compared to many traditional discrete distributions, DMPED offers several surprising benefits, especially when examining count data with high variation and that are positively skewed. We have explored several statistical characteristics of the assumed distribution, such as moments, the moment-generating function, the failure rate function, the monotonicity of the probability mass function, and a couple of descriptive measures (central tendency and dispersion). We have used the maximum likelihood estimation technique to estimate the parameters of the DMPED. We conducted a simulation study to validate the proposed estimators. Finally, four applications related to cancer diseases have been discussed, where DMPED (especially DMPEIID) fits the number of doses required for treatment, remission times, and therapy type comparisons.

## 1 Introduction

The majority of research and data used in medical applications. For example, the cell biological effects, such as repopulation, repair, redistribution, and re-oxygenation, based on the statistical model used. One model that examines these biological effects is the model using a Poisson distribution. This is the first model used to calculate the values of tumor control probability (TCP) and normal tissue complication probability (NTCP). For more information, see [1–4].

In addition, the Poisson distribution is a conventional distribution for describing count data with equal variance and mean, often known as equidispersion. However, in practical count data, the variation is usually greater than the mean, which is referred to as overdispersion, and only very rarely is the variance less than the mean, which is known as underdispersion. The Poisson distribution cannot explain these instances.

The discrete Erlang II distribution, introduced by Mosilhy, M.A. [5], is the best fit for these situations more than the Poisson distribution. The idea of combining the Poisson distribution with another continuous or discrete distribution to create a mixed distribution has received a lot of attention in the past, as the work of Greenwood and Yule [6] after which it was studied by Jonson et al. [7], but in recent years researchers seek applications of mixed distributions, as in [8,9] where they have examined the applications of the mixed Poisson distribution. The reader can also see some work on the properties of discrete Erlang distributions obtained by [10,11].

The purpose of this work is to use the sensitivity of the rate parameter for the proposed distribution in medical applications, such as applications that compare two therapies for leukemia, applications that calculate the number of doses (chemical or radiation therapy) in certain cancer diseases, and applications that provide information to patients.

In this study, we identified four different types of cancer datasets as interesting uses for *DMPEIID*. The first dataset, displays the number of doses required to cure 35 breast cancer patients, as well as the average number of doses required based on the estimator parameter is obtained. The second dataset, reviewed by [12], compares two approaches to the treatment of 101 patients with acute leukemia by transplanting their marrow. The third and fourth datasets [13,14], the likelihood of the tumor disappearing after therapy, the number of weeks that patients with tongue cancer had to remain during treatment, and the interval between remise and deaths are examples of results usually stated in terms of weeks in survival analysis.

The proposed distribution and the statistically significant functions related to the distribution are presented in Sect 2 of this work. We shall discuss the results of the suggested distribution method in Sect 3. In the first, this incorporated the distributional properties found in Sect 3.1. Additionally, the parameter estimator in the second is obtained by applying the maximum likelihood estimation approach. In Sect 3.2. We examine numerical and simulation research in the latter in Sect 3.3. We verify the proposed distribution in Sect 4 using four tumor cancer data sets, taking into account the dose, the comparison of two treatment approaches, the duration of the response and the duration of mortality. Finally, Sect 5 offers some concluding remarks on the investigation’s general findings.

## 2 Materials and methods

### 2.1 Erlang distribution

The age distribution of cancer incidence is usually based on the Erlang distribution; in addition, the shape and scale parameters show the number of driving events and the time interval between them and can be used to calculate the number of driving events for all forms of cancer. As in [15]. And multistage models contributed to the Erlang distribution being offered as a good approximation of the cell cycle time distribution in general, as in [16]. Also, Erlang [17] created it to forecast the number of simultaneous calls received by switching station operators. The distribution also applies to stochastic processes. The pdf for the Erlang distribution is

$$f_{E}(x;\gamma ,k)=\frac{\gamma^{k}x^{k-1}e^{-\gamma x}}{(k-1)!}\;\;\text{for}\;x,\gamma \geq 0\;\&\;k\geq 1.$$ (2.1)

*γ* is the rate parameter and *k* is the shape parameter.

The distribution function (cdf) within the Erlang distribution is

$$F_{E}(x;\gamma ,k)=1-\sum_{j=0}^{k-1}\left(\frac{(x\;\gamma {)}^{j}e^{-\gamma x}}{j!}\right).$$ (2.2)

### 2.2 Discrete mixed Poisson-Erlang distribution

When the characteristics of the data differ from what would be predicted by the simple component distribution, a number of distributions are commonly used to model the observed conditions. For example, observable data on the number of claims in actuarial or medical applications frequently exhibit a variance that clearly exceeds the mean. In such instances, it is unlikely that the claimed frequency distribution will have a Poisson shape.

In general, mixtures which are larger families of distributions are thought to be more adaptable than the models in use today, see [8,9].

Thus, in this study, we introduce a new discrete mixing of distributions, which we call the discrete mixed Poisson-Erlang distribution as follows:

A random variable *Z* follows the DMPED arising from mixing the Poisson distribution with the Erlang distribution, where the random variable Z~Poiss(λ) and *λ* is a random variable that tends to the Erlang distribution with pmf $f_{E}(\lambda ;\gamma ,k)$ in Eq 2.1. Now, we can derive the new pmf of the DMPED mixture as in [8], the pmf of DMPED is given as follows:

$$f_{DMPED}(z;\gamma ,k)=\int_{0}^{\infty }\frac{e^{-\lambda }\lambda^{z}}{z!}f_{E}(\lambda ;\gamma ,k)\;d\lambda \;=\int_{0}^{\infty }\frac{e^{-\lambda }\lambda^{z}}{z!}[\frac{\gamma^{k}\lambda^{k-1}e^{-\gamma \lambda }}{(k-1)!}]\;d\lambda \;=\frac{\gamma^{K}\;}{z!\;(k-1)!\;(\gamma +1{)}^{\left(z+k\right)}}\int_{0}^{\infty }(\lambda \;(\gamma +1){)}^{z+k-1}\;e^{-\lambda \;(\gamma +1)}\;d[\lambda \;(\gamma +1)]\;=\frac{\gamma^{K}}{z!\;(k-1)!\;(\gamma +1{)}^{\left(z+k\right)}}\;\Gamma (z+k)$$ (2.3)

where z=0,1,2,…;γ≥0;k≥1.

**Fig 1:**
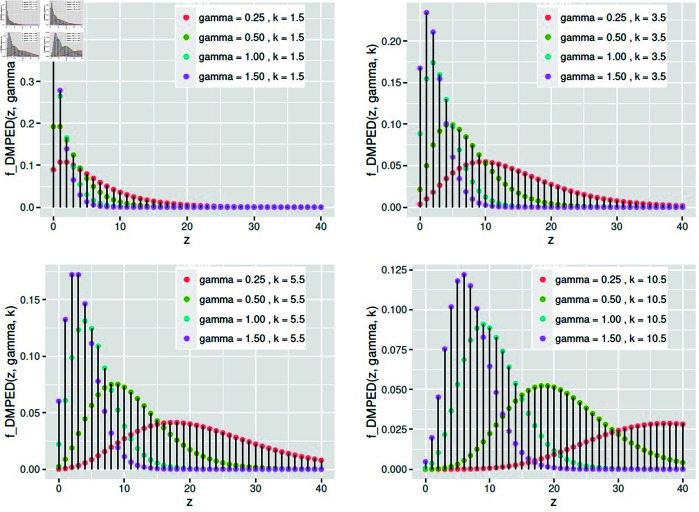
The pmf of *DMPED* for different values of *γ* and *k.*

**Fig 2:**
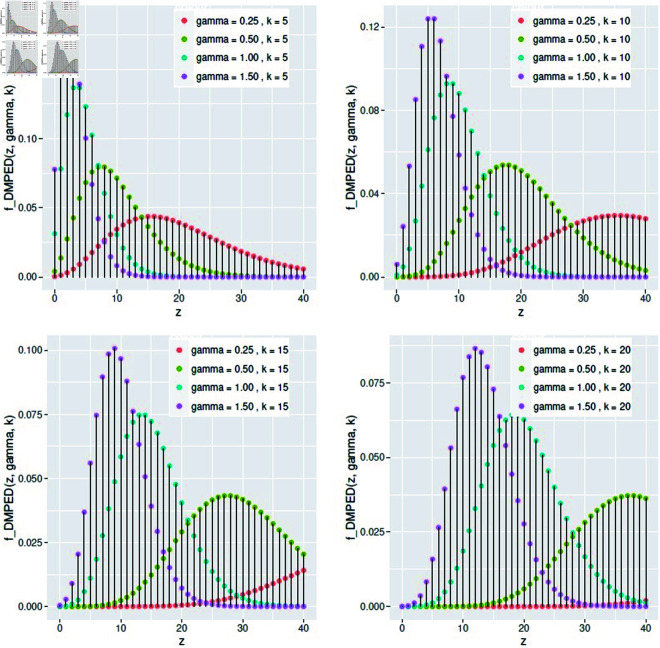
The pmf of *DMPED* for different values of *γ* and *k.*

We notice that there are two cases for the parameter *k* as follows:

1. **when *k* is rational number,** then *pmf* of the discrete mixed Poisson Erlang distribution (DMPED) in Eq (2.3) is
  $$f_{DMPED}(z;\gamma ,k)=\frac{\gamma^{K}\;\Gamma (z+k)}{z!\;\Gamma (k)\;(\gamma +1{)}^{\left(z+k\right)}},\;\;z=0,1,2,\ldots ;\;\gamma \geq 0;\;k\geq 1.$$ (2.4)
  Fig 1 shows the behavior of the pmf for DMPED with different values of *γ* and *k*.
2. **when *k* is a positive integer (i.e. k=1,2,…)** we obtain the discrete mixed Poisson Erlang-k distribution (DMPEKD) in Eq (2.3) is
  $$f_{DMPEKD}(z;\gamma ,k)=C_{k-1}^{z-1}\;(\gamma +1{)}^{-z}\;\gamma^{k},\;\;z=0,1,2,\ldots ;\;\gamma \geq 0.$$ (2.5)
  We notice that the DMPED in this case is similar to the negative binomial distribution. Fig 2 shows the behavior of the DMPED pmf under different values of *γ* and *k*, which is a positive integer.

#### 2.2.1 Behavior of the DMPED at Z tends to 0 and infinity.

Its important to study the behavior of the DMPED and Poisson distributions at *Z* = 0, and Z=∞, so its easy to prove that, for more information, you can see in [18,19]


$$\lim_{z\to 0}f_{\text{DMPED}}\text{(z;}\gamma \text{,k)}=\frac{\gamma^{k}}{(k-1)!}\lim_{z\to 0}\frac{\left(\gamma +1{)}^{-k-z}\Gamma (k+z\right)}{z!}\;=\left\{ \begin{array}{c} \frac{\gamma^{k}\;(\gamma +1{)}^{-k}\;\Gamma (k)}{(k-1)!}\;\;\text{for}\;\gamma \geq 0\;\&\;k\text{ is positive rational},\\ {\left(\frac{\gamma }{\gamma +1}\right)}^{k}\;\;\;\text{for}\;\gamma \geq 0\;\&\;k\text{ is positive integer.} \end{array}\right.$$

and


$$\lim_{z\to \infty }f_{\text{DMPED}}\text{(z;}\gamma \text{,k)}=\frac{\gamma^{k}}{(k-1)!}\lim_{z\to \infty }\frac{\left(\gamma +1{)}^{-k-z}\Gamma (k+z\right)}{z!}=0\;\text{for}\;\gamma \geq 0\;\&\;k\geq 1.$$

while the Poisson distribution has the following limits,


$$\lim_{z\to 0}f_{\text{Poiss}}(\text{z};\lambda )=e^{-\lambda }$$

$$\lim_{z\to \infty }f_{\text{Poiss}}(\text{z};\lambda )=0$$

**Fig 3:**
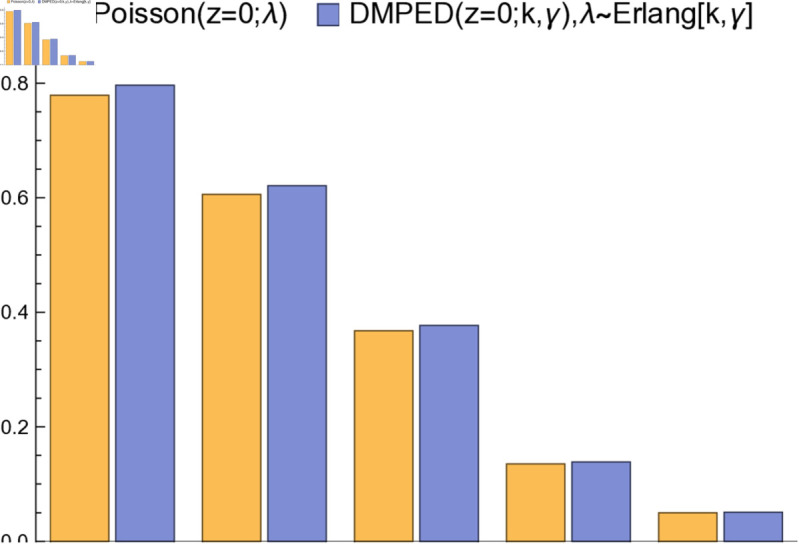
A comparison between DMPED and Poisson distribution at *Z* = 0 for different values of *λ.*

The bar chart in Fig 3 shows a comparison between the DMPED and the Poisson distribution at *z* = 0 for different values of λ., we can notice that the DMPED is greater than the Poisson distribution at *z* = 0.

### 2.3 Discrete mixed Poisson-Erlang - II distribution

The discrete mixed Poisson-Erlang II distribution (*DMPEIID*) is obtained by dealing with the particular situation where the shape parameter *k* = 2 and the rate parameter α=γ  +  1 in Eq (2.5). We can then deduce the pmf of *DMPEIID* as follows:

$$f_{DMPEII}(z;\alpha )=P_{r}(Z=z)=\alpha^{-z}(\alpha -1{)}^{2}\;(z-1),\;\;\alpha >1\;and\;z=2,3,4,\ldots$$ (2.6)

**Fig 4:**
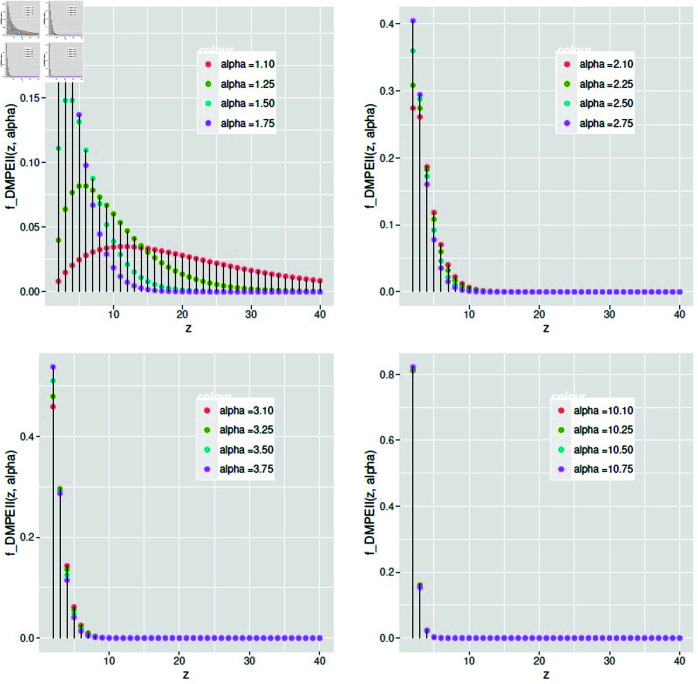
The pmf of *DMPEIID* for different values of *α.*

Fig 4 shows that the sensitivity of the rate parameter *α* causes a modification in the shape of the pmf at different values *α*, with a little difference between them. It is also observed that as the rate parameter increases, the mode of the distribution moves to the left.

The appropriate cdf for the *DMPEII* distribution is

$$F_{DMPEII}(z;\alpha )=P_{r}(Z\leq z)=\left\{ \begin{array}{c} 1-\alpha^{-z}(1+z\;(\alpha -1)),\;\;z\geq 2,\\ \;\;0,\;\;\;\;\text{Othewise}. \end{array}\right.$$ (2.7)

**Fig 5:**
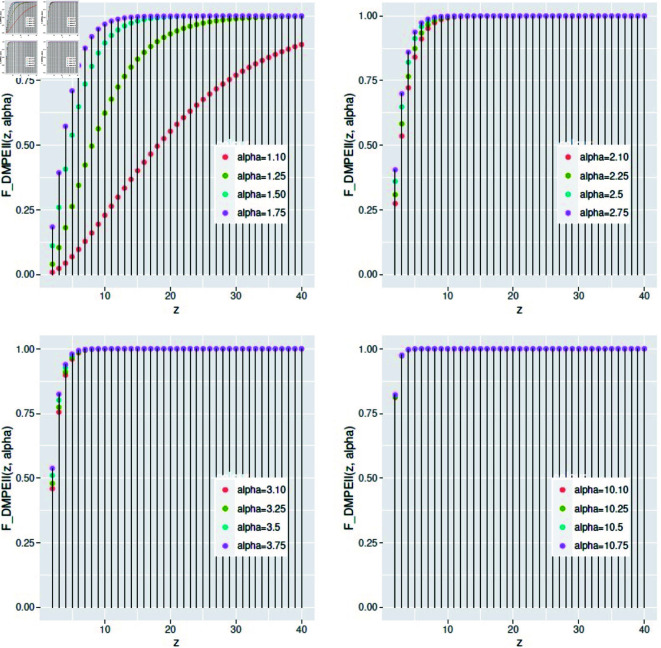
The cdf of *DMPEIID* with different *α* values.

Fig 5 shows that the sensitivity of the rate parameter *α* influences the shape of the cumulative distribution function (cdf) at various values with minimal variation. As *α* increases, the probability of obtaining high values also increases, suggesting that the cumulative distribution function approaches rapidly.

The subsequent subsections delineate the reliability and Mills ratio functions, together with the discrete hazard rate (*hr*) and reverse hazard rate (*rhr*) functions; see [20,21].

#### 2.3.1 Reliability function of discrete Mixed Poisson-Erlang II distribution.

The analytical structure of the *DMPEII* distribution makes it an effective instrument to define the duration of the failure of a system. The reliability function (Survival function), which represents a system’s ability to work under pertinent conditions prior to a specified value *z*, is represented as *R*(*z*). Consequently, the *R*(*z*) function of a *DMPEII* distribution is provided by

$$R_{DMPEII}(z;\alpha )=1-F_{DMPE_{2}}(z;\alpha )=\alpha^{-z}(1+z\;(\alpha -1));\;\alpha >1,\;z=2,3,4,\ldots$$ (2.8)

**Fig 6:**
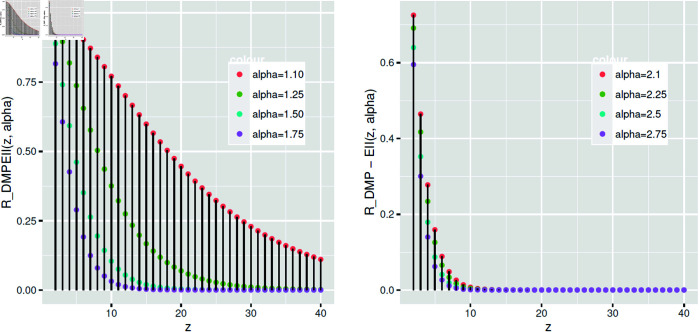
*DMPEIID*’s reliability function varies with *α.*

According to Fig 6, the reliability function of *DMPEII* grows when *α* rises at different rates.

#### 2.3.2 Hazard rate function of discrete Mixed Poisson-Erlang II distribution.

The *DMPEII* distribution’s hazard rate (*hr*) function is often stated as follows:

$$hr_{DMPEII}(z;\alpha )=\frac{f_{DMPEII}(z;\alpha )}{S_{DMPEII}(z;\alpha )}\;=\frac{\left(\alpha -1{)}^{2}(z-1\right)}{z\alpha (\alpha -1)-\alpha^{2}+2\alpha };\;\alpha >1,\;z=2,3,4,\ldots$$ (2.9)

**Fig 7:**
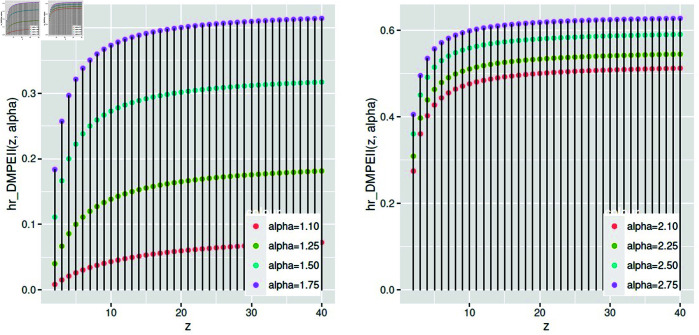
The hazard rate function of *DMPEIID* at different values of *α.*

The *hr* function of *DMPEIID* improves as *α* climbs at different rates, as Fig 7 illustrates.

#### 2.3.3 The reverse hazard rate function of discrete Mixed Poisson-Erlang II distribution.

The reverse hazard rate (*rhr*) function of the *DMPEII* distribution is often defined as follows:

$${rhr}_{DMPEII}(z;\alpha )=\frac{f_{DMPEII}(z;\alpha )}{F_{DMPEII}(z;\alpha )}\;=\frac{\left(\alpha -1{)}^{2}(z-1\right)}{\alpha^{z}-z(\alpha -1)-1};\;\alpha >1,\;z=2,3,4,\ldots$$ (2.10)

**Fig 8:**
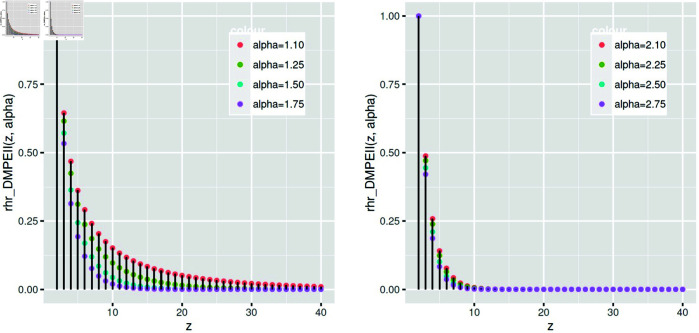
The reverse hazard rate function of *DMPEIID* varies according to *α.*

Fig 8 shows that increasing the rate parameter *α* reduces the *rhr* function of *DMPEIID*.

#### 2.3.4 Mills Ratio of discrete Mixed Poisson-Erlang II distribution.

We may obtain Mills Ratio (*MR*) of the *DMPEII* distribution as follows:

$${MR}_{DMPEII}(z;\alpha )=\frac{1}{{rhr}_{DMPEII}(z;\alpha )}\;=\frac{\alpha^{z}-z(\alpha -1)-1}{\left(\alpha -1{)}^{2}(z-1\right)};\;\alpha >1,\;z=2,3,4,\ldots$$ (2.11)

**Fig 9:**
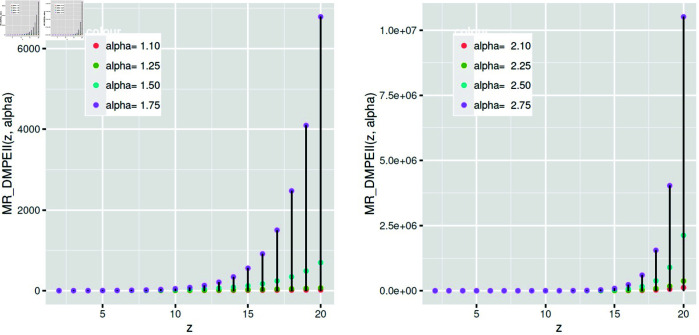
The Mills Ratio function of *DMPEII* depends on *α.*

The function *MR* of *DMPEIID* is shown in Fig 9 for various values of the rate parameters.

## 3 Results

The main features of the proposed distribution will be presented in three parts of this section.

### 3.1 Distributional characteristics

Several stochastic properties of the previously described distribution, *DMPEII*, are inferred in this subsection of the paper.

#### 3.1.1 Quantile function for discrete Mixed Poisson-Erlang II distribution.

**Theorem 3.1** *Given a random variable z of DMPEII distribution with the parameter *α*, then the r^the^ Quantile function (Qf) is given as*

$$Q_{DMPEII}(r;\alpha )=\log_{\alpha }[\frac{1}{1-r}];\;\alpha >1$$ (3.1)

*3.1.1.1 Proof:*

By inverting the cdf in Eq (2.7), the quantile of order 0 < *r* < 1 could be derived as follows


$$F_{DMPEII}(Q;\alpha )=\{1-\alpha^{-Q}[1+Q\;(\alpha -1)]\};\;\alpha >1,\;Q=2,3,\ldots$$

Then $F_{DMPEII}^{-1}(r;\alpha )=min\{x\epsilon R:F_{DMPEII}(x;\alpha )\geq r\}$ that is


$$r=1-\alpha^{-Q}\;[1+Q\;(\alpha -1)]$$

$$\left(1-r)=\alpha^{-Q}\;[1+Q\;(\alpha -1)\right]$$

$$\log_{\alpha }(1-r)=-Q+\log_{\alpha }\left[1+Q\;(\alpha -1)\right]$$

Now, take $\;u=\log_{\alpha }\left[1+Q\;(\alpha -1)\right]\Rightarrow Q=\frac{\alpha^{u}-1}{\alpha -1}$, and so


$$u-\frac{\alpha^{u}-1}{\alpha -1}=\log_{\alpha }(1-r)\;\Rightarrow \;u(\alpha -1)-\alpha^{u}=\log_{\alpha }(1-r{)}^{\left(\alpha -1\right)}-1$$

Therefore, from properties of logarithm we get


$$u=\log_{\alpha }\frac{\alpha }{(1-r{)}^{\alpha -1}}$$

Moreover,


$$\log_{\alpha }\left[1+Q\;(\alpha -1)\right]=\log_{\alpha }\frac{\alpha }{(1-r{)}^{\alpha -1}}\;\Rightarrow \;1+Q\;(\alpha -1)=\frac{\alpha }{(1-r{)}^{\alpha -1}}$$

And so,


$$Q=\log_{\alpha }[\frac{1}{1-r}]$$

So, the *r^th^* Quantile function (Qf) is


$$Q_{DMPEII}(r;\alpha )=\log_{\alpha }[\frac{1}{1-r}]$$

So the theorem is proved. □

Note that the first quartile, the median and the third quartile of the *DMPEII* distribution can be computed by substituting by r=0.25,r=0.5 and *r* = 0.75 in Eq (3.1). And so, the Inter Quartile Range is equal to


$$IQR=Q_{{DMPEII}_{3}}(0.75;\alpha )-Q_{{DMPEII}_{1}}(0.25;\alpha )=\log_{\alpha }[3]$$

#### 3.1.2 Moments and moment generating function for discrete Mixed Poisson-Erlang II distribution.

In this subsection, the moments and moments generating function (*mgf*) for a random variable *z* with the *DMPEII* distribution and parameter *α* are generated by the below theorem.

**Theorem 3.2** *Given a random variable z with the DMPEII distribution, its moment generating function (mgf) is*

$$M_{z}(t)=[\frac{\alpha -1}{\alpha e^{-t}-1}{]}^{2}$$ (3.2)

*where $\alpha >1\;and\;\alpha >e^{t}$.*

*3.1.2.1 proof:*

Using the traditional interpretation of the moment generating function, we obtain


$$M_{z}(t)=E(e^{zt})=\sum_{allz}e^{tz}f_{DMPEII}(z;\alpha )$$

$$=\sum_{z=2}^{\infty }e^{tz}[\alpha^{-z}(\alpha -1{)}^{2}\;(z-1)]$$

$$=(\alpha -1{)}^{2}\sum_{z=2}^{\infty }e^{tz}\alpha^{-z}\;(z-1)$$

$$=(\alpha -1{)}^{2}\sum_{z=2}^{\infty }[\alpha e^{-t}{]}^{-z}(z-1)$$

$$=[\frac{\alpha -1}{\alpha e^{-t}-1}{]}^{2};\;\;\alpha >1\;and\;\alpha >e^{t}$$

So the theorem is proved. □

Thus, we can obtain the mean (which equal to the first moment about the origin) of the *DMPEII* distribution as follows by applying Eq (3.2):

$$m_{1}=E(Z)=\frac{dM_{Z}(t)}{dt}{|}_{t=0}=\frac{2\alpha }{\left(\alpha -1\right)},\;\;\alpha >1.$$ (3.3)

Also, the second moment on the origin of the *DMPEII* distribution is

$$m_{2}=E(Z^{2})=\frac{d^{2}M_{Z}(t)}{dt^{2}}{|}_{t=0}=\frac{2\alpha +4\alpha^{2}}{(\alpha -1{)}^{2}},\;\;\alpha >1.$$ (3.4)

So, the variance of the *DMPEII* distribution using Eqs (3.3) and (3.4) is

$$\sigma_{DMPEII}^{2}=m_{2}-(m_{1}{)}^{2}\;=\frac{2\alpha }{(\alpha -1{)}^{2}},\;\;\alpha >1.$$ (3.5)

Thus, the standard deviation of the *DMPEII* distribution is

$$\sigma_{DMPEII}=\frac{\sqrt{2\alpha }}{\alpha -1},\;\;\alpha >1.$$ (3.6)

Consequently, the coefficient of variation of the *DMPEII* distribution is

$${C.Var}_{DMPEII}=\frac{\sigma }{m_{1}}=\sqrt{\frac{\alpha }{2}},\;\;\alpha >1.$$ (3.7)

The following relation yields the *r^the^* moment about the origin of the *DMPEII* distribution:

$$m_{r}=E(Z^{r})=\frac{d^{r}M_{Z}(t)}{dt^{r}}{|}_{t=0}.$$ (3.8)

For instance, the third moment concerning the origin of the *DMPEII* distribution is


$$m_{3}=E(Z^{3})=\frac{d^{3}M_{Z}(t)}{dt^{3}}{|}_{t=0}$$

$$=\frac{2\alpha (4\alpha^{4}+7\alpha +1)}{(\alpha -1{)}^{3}},\;\;\alpha >1.$$

and so on.

#### 3.1.3 Probability generating function for discrete Mixed Poisson-Erlang II distribution.

In this subsection, probability generating (*pgf*) for a random variable *z* with the *DMPEII* distribution and parameter *α* are generated.

**Theorem 3.3.** *Given a random variable z with the DMPEII distribution, its probability generating function (pgf) is*

$$G_{z}(t)=[\frac{t(\alpha -1)}{\alpha -t}{]}^{2}$$ (3.9)

*where α>1andα≠t.*

*3.1.3.1 Proof:*

Using the traditional interpretation of the probability generating function, we get


$$G_{z}(t)=E(t^{z})=\sum_{allz}t^{z}f_{DMPEII}(z;\alpha )$$

$$=\sum_{z=2}^{\infty }t^{z}[\alpha^{-z}(\alpha -1{)}^{2}\;(z-1)]$$

$$=(\alpha -1{)}^{2}\sum_{z=2}^{\infty }(\frac{\alpha }{t}{)}^{-z}\;(z-1)$$

$$=[\frac{t(\alpha -1)}{\alpha -t}{]}^{2};\;\;\alpha >1\;and\;\alpha \neq t$$

So the theorem is proved. □

As a result, using Eq (3.9), we can get the mean (the first moment about the origin) of the *DMPEII* distribution as follows:

$$m_{1}=E(Z)=\frac{dG_{Z}(t)}{dt}{|}_{t=1}=\frac{2\alpha }{\left(\alpha -1\right)},\;\;\alpha >1.$$ (3.10)

Thus, employing Eq (3.10) and the conventional interpretation of (pgf), the variance of the *DMPEII* distribution is

$$\sigma_{DMPEII}^{2}=\frac{d^{2}G_{Z}(t)}{dt^{2}}{|}_{t=1}+m_{1}-[m_{1}{]}^{2}\;=\frac{2\alpha }{(\alpha -1{)}^{2}},\;\;\alpha >1.$$ (3.11)

Notably, the results of the mean and variance, which are determined by (mgf) and (pgf), are equivalent.

#### 3.1.4 Monotonicity for discrete Mixed Poisson-Erlang II distribution.

Studying the monotonic growing (or decreasing) of pmf and the monotonic increasing (or decreasing) of the hazard rate function for the *DMPEII* in the following two scenarios is crucial:

1. **Monotonicity of pmf** As shown below, we first set the first derivative of pmf to zero to find the critical point for the monotonicity of pmf in the *DMPEII* distribution.
  $$f_{DMPEII}^{\prime }(z;\alpha )=(\alpha -1{)}^{2}\alpha^{-z}(1-(z-1)\ln\alpha ),$$ (3.12)
  Put $f_{DMPEII}^{\prime }(z;\alpha )=0$ to get
  $$f_{DMPEII}^{\prime }(z_{0};\alpha )=0\Rightarrow z_{0}=1+\frac{1}{\ln\alpha }>1$$ (3.13)
  So, the pmf of *DMPEII* is monotonically decreasing for all values of *Z*, since α>1. Reproduced from Fig 4.
2. **Monotonicity of hazard rate function** It is necessary to investigate the monotonic behavior of the risk rate function, as seen in [20]. Use the same test as before to accomplish this:
  $$h_{DMPEII}^{\prime }(z;\alpha )=\frac{\alpha (\alpha -1{)}^{2}}{(1+z(\alpha -1){)}^{2}}.$$
  The *hr* function shows a monotonic increase for all values of *Z* and *α*. Because $h_{DMPEII}^{\prime }(z;\alpha )>0;$ ∀z=2,3,…. Fig 5 shows how the function *hr* grows with different values of the rate parameter *α*.

#### 3.1.5 Mode, skewness, kurtosis, and the Fano factor of discrete Mixed Poisson-Erlang II distribution.

This subsection contains results regarding the mode, skewness, kurtosis, Fano factor, and other critical metrics (central tendency and dispersion). Discrete distribution is essential in a variety of fields, including medical statistics.

1. **The mode** In the first, we derive the critical point of pmf for the distribution *DMPEII* by calculating the first derivative, which we introduce in Eq (3.12) and use Eq (3.13), obtaining the point *z*_0_ = 1  +  $\frac{1}{\ln\alpha }$. To ascertain if this point represents the local maximum (minimum), compute the second derivative of $f_{DMPEII}(z;\alpha )$:
  $$f_{DMPEII}^{\prime \prime }(z_{0};\alpha )=\frac{\ln\alpha^{-(\alpha -1{)}^{2}}}{e\alpha },\;\alpha >1$$
  Since $f_{DMPEII}^{\prime \prime }(z_{0};\alpha )<0;\;\forall \alpha$, $\;z_{0}\;$ is the local maximum point of pmf, which means that the mode of *DMPEII* distribution is equal to
  $$Mode_{DMPEII}=z_{0}=1+\frac{1}{\ln\alpha }.$$ (3.14)
  Consequently, the distribution of *DMPEII* is umimodal.
2. **The skewness** To measure a probability distribution’s inadequate symmetry, a number of skewness metrics have been proposed in the literature. The most widely used of these is Karl Pearson’s measure, which can be written by the following formula:
  $$SK_{DMPEII}=\frac{Mean-Mode}{\text{Standard Deviation}}=\frac{m_{1}-Mode_{DMPEII}}{\sigma_{DMPEII}}.$$
  Using Eqs (3.3), 3.6) and (3.14) we get the coefficient of skewness of *DMPEII* distribution is
  $$SK_{DMPEII}=\frac{(1-\alpha )+(1+\alpha )\ln\alpha }{\sqrt{2\alpha }\;\ln\alpha },\;\alpha >1.$$ (3.15)
3. **The kurtosis** By applying the traditional meaning of the coefficient of kurtosis, which is
  $$kur_{DMPEII}=\frac{\mu_{4}}{\mu_{2}^{2}}=\frac{\mu_{4}}{(variance{)}^{2}}$$
  Where $\mu_{4}$ is the fourth moment about the mean and $\mu_{2}$ is the second moment about the mean (the variance). Using the wolfram mathematica programming, we get
  $$\mu_{4}=\sum_{z=2}^{\infty }\left(z-m_{1}{)}^{4}f_{DMPEII}(z;\alpha \right)=\frac{2\alpha (\alpha^{2}+10\alpha +1)}{(\alpha -1{)}^{4}}$$ (3.16)
  By using Eq (3.5) and Eq (3.16), we obtained
  $$kur_{DMPEII}=\frac{\alpha^{2}+10\alpha +1}{2\alpha },\;\;\alpha >1.$$ (3.17)
4. **Fano factor** Similar to the coefficient of variation, the Fano factor (dispersion index) is a statistical indicator of the dispersion of a probability distribution containing Fano noise. It is named after the Italian American physicist Ugo Fano (1974), for more information (you can see [22]). The “Fano factor” (*FF*_*DMPEII*_), using Eqs (3.3) and (3.5), is given by
  $$FF_{DMPEII}=\frac{Variance}{Mean}=\frac{\sigma_{DMPEII}^{2}}{m_{1}}=\frac{1}{\alpha -1},\;\;\alpha >1.$$ (3.18)
  In ecology, it’s commonly used as a standard measure for determining repulsion (under dispersion) or clustering (over dispersion), depending on whether a given model is appropriate for over- or under-dispersed datasets. The distribution is over-dispersed if the Fano factor *FF*_*DMPEII*_>1, while it will be under-dispersed if the vlaue *FF*_*DMPEII*_<1.

Table 1 provides descriptive statistics of the *DMPEII* distribution at various parameter levels. It is evident that the skewness and kurtosis gradually surpass when the form parameter settings increase. The proposed distribution is successful for over-scattered (under-scattered) data when *FF*_*DMPEII*_>1(<1). The other observation is that the variance is higher than the mean for 1<α<2, while the reverse is true for when α>2.

**Table 1:** *Mean*, *Mode*, Variance (*Var*.), Skewness (*SK*), Kurtosis (*Kur*), and the Fano Factor (*FF*) of *DMPEII* for various α values at n=50.

*α*	*Mean*	*Mode*	*Var*.	*SK*	*kur*	*FF*
1.10	19.80	11.49	129.4	0.73	2.63	6.53
1.25	9.99	5.48	39.61	0.72	5.59	3.96
1.50	6.00	3.47	11.99	0.73	6.08	2.00
1.75	4.67	2.79	6.22	0.75	6.16	1.33
2.10	3.82	2.35	3.47	0.79	6.29	0.91
2.25	3.60	2.23	2.88	0.81	6.35	0.80
2.50	3.33	2.09	2.22	0.83	6.45	0.67
2.75	3.14	1.99	1.79	0.86	6.56	0.57
3.10	2.95	1.88	1.41	0.90	6.71	0.48
3.25	2.89	1.85	1.28	0.92	6.78	0.44
3.50	2.80	1.79	1.12	0.95	6.89	0.40
3.75	2.73	1.76	0.99	0.98	7.01	0.36

### 3.2 Maximum likelihood estimation for discrete Mixed Poisson-Erlang II distribution

The goal of this section is to determine the parameters of the suggested *DMPEII* distribution using a maximum likelihood estimate (MLE).

For a random sample of size n with the *DMPEII* distribution, $Z_{1},Z_{2},\ldots ,Z_{n}$, the likelihood function is given by


$$L_{DMPEII}(z_{i},\alpha )=\prod_{i=1}^{n}f_{DMPEII}(z_{i};\alpha )=\prod_{i=1}^{n}[\alpha^{-z_{i}}(\alpha -1{)}^{2}\;(z_{i}-1)];\;\;\alpha >1$$

The log-likelihood function is given by

$$l_{DMPEII}(z_{i},\alpha )=\ln\left[L_{DMPEII}(z_{i},\alpha )\right]\;=\sum_{i=1}^{n}\ln\left[\alpha^{-z_{i}}(\alpha -1{)}^{2}\;(z_{i}-1)\right]\;=n\ln\left[\frac{(\alpha -1{)}^{2}}{e}\right]+\sum_{i=1}^{n}[z_{i}\ln\left(\frac{e}{\alpha }\right)];\;\;\alpha >1$$ (3.19)

Differentiating Eq (3.19). Given the parameter *α*, we obtain the following equation:

$$\frac{d\;l_{DMPEII}(z_{i},\alpha )}{d\;\alpha }=\frac{2n}{\alpha -1}-\frac{\sum_{i=1}^{n}z_{i}}{\alpha }$$ (3.20)

Now, putting


$$\frac{d\;l_{DMPEII}(z_{i},\alpha )}{d\;\alpha }=0,$$

$$\frac{2\alpha }{\alpha -1}=\frac{\sum_{i=1}^{n}z_{i}}{n};\;\alpha >1$$ (3.21)

Since


$$\frac{\sum_{i=1}^{n}z_{i}}{n}=\bar{Z}$$

is the mean of $\;Z_{1},Z_{2},\ldots ,Z_{n}$. So we get

$$\frac{2\alpha }{\alpha -1}=\bar{Z};\;\alpha >1$$ (3.22)

Eq (3.22) yields MLE for the parameter $\hat{\alpha }$.

$$\hat{\alpha }=(\frac{\bar{Z}}{\bar{Z}-2});\;\bar{Z}\neq 2$$ (3.23)

If $\;\bar{Z}=2$, then from Eq (3.22), we get


$$\frac{2\alpha }{\alpha -1}=2,$$

Which is theoretically impossible.

### 3.3 Studies of Numerical and Simulation for discrete Mixed Poisson-Erlang II distribution

This subsection presents the Monte Carlo simulation that was run with 5,000 replications to test the accuracy of point estimates for the parameter of the suggested *DMPEII* distribution and its related reliability and hazard rate functions using MLE. For further reading on simulation studies, one may refer to the work in [23–25].

The simulation was carried out with sample sizes of n = 10, 30, 50, 100 and 150, and some different values for the parameter *α*. The average (mean) estimate (AE) and the mean squared error (MSE) were employed as criteria measures to assess the point estimation of the parameter of the *DMPEII* distribution.

To generate sample data from DMPEII(α) we solve the following equation:


1−u=R(z;α),

and


$$z=R^{-1}(1-u;\alpha ),\;u\sim Uniform(0,1)$$

where $R^{-1}(\_;\alpha )$ is the inverse of the reliability function in 2.8.

Table 2 shows the AE and MSE of the simulation for *α* with assumed different true values as (1.1,1.5,1.75,2,2.5,3), while Tables 3 and 4 show the AE and MSE for the hazard rate function h(t,α) and reliability function R(t,α) at time $t=m_{1}=E(z;\alpha )$ based on different true values of *α* with different sample sizes of *n*.

**Table 2:** AE and MSE for the ML estimates of α based on different values of n.

*α*	n	${\hat{\alpha }}_{ML}$				
		10	30	50	100	150
1.1	AE	1.1057	1.1017	1.1010	1.1007	1.1003
	MSE	7.35E-04	1.99E-04	1.15E-04	5.79E-05	3.60E-05
1.5	AE	1.5429	1.5132	1.5070	1.5029	1.5027
	MSE	2.85E-02	7.49E-03	4.07E-03	1.94E-03	1.30E-03
1.75	AE	1.8185	1.7754	1.7650	1.7562	1.7551
	MSE	8.66E-02	2.00E-02	1.13E-02	4.89E-03	3.31E-03
2	AE	2.1194	2.0342	2.0189	2.0074	2.0055
	MSE	2.07E-01	4.10E-02	2.15E-02	1.07E-02	6.75E-03
2.5	AE	2.7222	2.5785	2.5307	2.5180	2.5103
	MSE	9.15E-01	1.27E-01	6.18E-02	3.08E-02	1.97E-02
3	AE	3.4285	3.0949	3.0524	3.0363	3.0245
	MSE	2.43E+00	2.71E-01	1.43E-01	6.45E-02	4.23E-02

**Table 3:** AE and MSE for the ML estimates of the hazard function hr(z,α) based on different values of n.

${\hat{hr}}_{DMPEII}({\hat{m}}_{1};\hat{\alpha })$							
*α*	t	n	10	30	50	100	150
1.1	22	AE	0.0677	0.0641	0.0637	0.0639	0.0636
		MSE	4.06E-04	1.07E-04	6.64E-05	3.40E-05	2.36E-05
1.5	6	AE	0.2727	0.2636	0.2616	0.2608	0.2609
		MSE	4.78E-03	1.38E-03	8.16E-04	4.04E-04	2.78E-04
1.75	4.67	AE	0.3609	0.3519	0.3500	0.3473	0.3476
		MSE	6.60E-03	2.05E-03	1.21E-03	5.76E-04	3.75E-04
2	4	AE	0.4327	0.4205	0.4191	0.4170	0.4165
		MSE	8.48E-03	2.46E-03	1.44E-03	7.77E-04	4.93E-04
2.5	3.33	AE	0.5312	0.5236	0.5217	0.5206	0.5216
		MSE	9.79E-03	3.36E-03	2.03E-03	9.12E-04	5.79E-04
3	3	AE	0.6154	0.5972	0.5964	0.5932	0.5944
		MSE	1.17E-02	3.33E-03	2.19E-03	9.53E-04	7.16E-04

**Table 4:** AE and MSE for the ML estimates of Reliability function R(z,α) based on different values of n.

${\hat{R}}_{DMPEII}({\hat{m}}_{1};\hat{\alpha })$							
*α*	t	n	10	30	50	100	150
1.1	22	AE	0.3809	0.3933	0.3937	0.3911	0.3923
		MSE	1.34E-02	4.49E-03	2.86E-03	1.47E-03	1.05E-03
1.5	6	AE	0.3405	0.3472	0.3493	0.3495	0.3488
		MSE	1.34E-02	4.61E-03	2.85E-03	1.43E-03	9.83E-04
1.75	4.67	AE	0.3261	0.3267	0.3280	0.3309	0.3301
		MSE	1.23E-02	4.50E-03	2.73E-03	1.33E-03	8.61E-04
2	4	AE	0.3031	0.3109	0.3112	0.3131	0.3134
		MSE	1.26E-02	4.27E-03	2.54E-03	1.39E-03	8.95E-04
2.5	3.33	AE	0.2798	0.2820	0.2830	0.2831	0.2816
		MSE	1.20E-02	4.56E-03	2.80E-03	1.28E-03	8.10E-04
3	3	AE	0.2442	0.2569	0.2568	0.2594	0.2578
		MSE	1.25E-02	4.02E-03	2.67E-03	1.20E-03	8.92E-04

All results in Tables 2, 3 and 4, explain that the estimates are close to the true values of the parameters for all sample sizes. Moreover, as the sample size increases, the MSEs decrease as expected, which means that the maximum likelihood method is appropriate to estimate the parameter and reliability functions of the *DMPEII* distribution.

**Fig 10:**
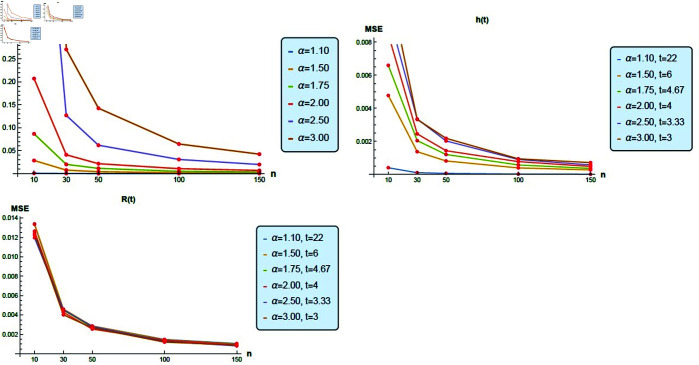
Comparison of the MSE for *α*, *hr*(*t*), and *R*(*t*) based on simulation results.

Fig 10 is constructed to compare the MSE for $\hat{\alpha }$, $\hat{h}(t)$ and $\hat{R}(t)$ based on a simulation study in which different values of *α*, *n* and *t* are assumed.

Based on this graph and the simulation tables, we are able to derive the following conclusions.

- As the sample size increases, the MSE decreases.
- The simulation results involving *α* and *hr*(*t*) show a direct correlation between the assumed values of *α* and MSE.
- For all values of *α*, the MSE of *R*(*t*) is very close to each other.

## 4 Applications

The proposed distribution has different merits, where it has only one parameter with high sensitivity and is also the best fit for applications that exhibit excess dispersion, a right skewness, and when the Fano factor is greater than one (this means that the variance is larger than the mean). The Fano factor is a crucial metric in therapeutic trials. In this part, we will examine whether fitted to four real lifetime counting datasets the proposed distribution, DMPEII, is adequate.

The distribution *DMPEII* is contrasted with fifth similar distributions to assess its effectiveness and ensure its goodness of fit: the discrete Burr distribution (*DBurr*) [26], the geometric distribution (*Geo*) [21], the discrete generalized exponential distribution of a second type (*DGE*_2_) [27], the discrete generalized Poisson distribution (*GPoiss*) [28,29], and the Poisson distribution (*Poiss*) [30].

Several statistical methods are available to determine the fitness of competing distributions, where the most widely used are the Akaike information criteria (AIC) (see Akaike [31]) and the Bayesian knowledge criteria (BIC) (see Schwarz [32] for more information). The optimal distribution for the real data set may be the one with the lowest values. Also, the Kolmogorov-Smirnov (*K*–*S*) test and its P-value (see [33] for additional information) have been applied. The model with the highest p-value and the lowest *K*–*S* value is considered optimal.

The summary findings for the three datasets are calculated in Table 5 using the central tendency and dispersion measures (minimum (*Min*), maximum (*Max*), mean (*Mean*), median (*Med*), mode (*Mode*), variance (*Var*.) skewness (*SK*), kurtosis (*Kur*.) and Fano factor (*FF*). Because *SK*>0, all datasets are clearly right-skewed, and *FF* > 1, they are excessively distributed.

**Table 5:** Summary Statistics for (Dataset1), (Dataset2-G1), (Dataset2-G2), (Dataset3) and (dataset4).

Datasets	n	Min	Max	Mean	Med.	*Mode*	*Var*.	*SK*	*Kur*.	*FF*
dataset1	35	1	50	15.7	12	6	173.9	1.2	3.3	11.1
dataset2-G1	50	1	61	15.6	14	2	147.9	1.1	4.9	8.8
dataset2-G2	51	1	57	16.5	14	3	143.7	1.1	4.2	9.2
dataset3	30	1	30	7.97	5.5	2	55.8	1.4	4.3	7.01
dataset4	30	1	35	13.8	9	4	120.9	0.6	1.8	8.8

### 4.1 Dataset1

The first dataset (dataset1) in Table 6 is a simulated data obtained from the DMPEII distribution.It can represent the total number of chemotherapy doses required for the 35 patients to recover from breast cancer.

**Table 6:** The total dosage required for the 35 patients in the group to recover from their tumors (dataset1).

1	4	4	4	6	6	6	6	6	6	6	6
6	8	8	9	9	12	12	12	13	15	15	18
18	18	19	22	30	35	35	39	40	45	50	

**Fig 11:**
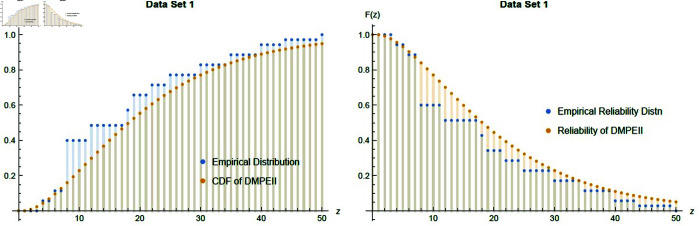
Empirical fitting graph of cdf and reliability between Dataset1 and *DMPEII* distribution.

Fig 11 indicates that there is no difference between the empirical distribution function of Dataset1 and *DMPEIID*. Additionally, the empirical reliability function is compatible with the *DMPEII* reliability function based on the MLE of the parameter.

However, Table 7 shows that the *DMPEII* distribution is the best-fit distribution for modeling dataset 1 when compared with other competitive distributions.

**Table 7:** Parameters estimates, -log (L), AIC, BIC, (*K*–*S*) and P-value (p-v) of *DMPEII*, *DBuur*, *DGPoiss*, *DGeo*, ${DGE}_{2}$, and *DPoiss* distributions for (dataset1).

Dist.	Parameters Estimates		-log(L)	AIC	BIC	*K*–*S*	p-v
*DMPEII*	$\hat{\alpha }=1.13$	–	**129.8**	**261.6**	**263.2**	**0.15**	**0.46**
*DBurr*	$\hat{\alpha }=70.3$	$\hat{\theta }=0.99$	153.4	310.7	313.8	0.4	2.6e-05
*DGPoiss*	$\hat{\lambda }=4.7$	$\hat{p}=0.69$	129.9	261.7	264.8	0.5	0.00017
*DGeo*	–	$\hat{p}=0.06$	132.4	266.9	268.4	0.5	0.00017
*DGE*_2_	$\hat{\alpha }=1.95$	$\hat{p}=0.9$	129.8	262.7	265.8	0.5	0.00018
*DPoiss*	$\hat{\lambda }=15.7$	–	242.09	486.2	487.8	0.5	0.00019

Finally, the average number of doses required to recover from a breast cancer tumor is stated as follows:


$${\hat{m}}_{1}=E(Z){|}_{\alpha =\hat{\alpha }}=\frac{2\hat{\alpha }}{\hat{\alpha }-1}{|}_{\hat{\alpha }=1.1}=22$$

### 4.2 Dataset2

The numerical data provided by [12] is extensively reviewed. A sample of 101 patients with advanced acute myelogenous leukemia who were registered with the International Bone Marrow Transplant Registry is shown in Tables 8 and 9. In order to replenish their compromised immune systems, fifty-one of these patients underwent autologous (auto) bone marrow transplants, in which their own marrow was reinfused after intensive chemotherapy. In order to restore their immune systems, fifty patients underwent an allogeneic (allo) bone marrow transplant, in which bone marrow from a sibling who matched them for HLA (Histocompatibility Leukocyte Antigen) was used.

The leukemia-free reliability periods (measured in months) for the first group (G1), 50 allogeneic transplant patients, are as follows

**Table 8:** The leukemia free-reliability times (in months) for the 50 allogeneic transplant patients (dataset2 - G1).

1	2	2	2	2	2	3	3	4	5
6	7	8	9	11	11	11	12	12	12
12	13	13	13	14	14	15	16	17	17
18	19	20	21	21	23	23	24	25	25
27	29	29	30	30	32	35	39	40	61

**Table 9:** The leukemia free-reliability times (in months) for the 51 autologous transplant patients (dataset2 - G2).

1	2	2	3	3	3	3	3	3	3	3
5	5	6	7	7	9	9	10	11	12	13
13	13	13	14	14	14	15	15	16	16	17
17	18	18	18	19	19	24	28	28	29	31
32	33	33	35	36	37	57				

For the second group (G2) of 51 autologous transplant patients, the leukemia free reliability durations (measured in months) are as follows

Tables 10 and 11 show the results for both groups in this dataset.

**Table 10:** Parameters estimates, -log (L), AIC, BIC, (*K*–*S*) and P-value (p-v) of *DMPEII*, *DBuur*, *DGPoiss*, *DGeo*, ${DGE}_{2}$, and *DPoiss* distributions for (dataset2-G1).

Dist.	Parameters Estimates		-log(L)	AIC	BIC	*K*–*S*	p-v
*DMPEII*	$\hat{\alpha }=1.1$	–	**188.36**	**378.73**	**380.64**	**0.13**	**0.37**
*DBurr*	$\hat{\alpha }=60.98$	$\hat{\theta }=0.99$	223.86	451.71	455.53	0.33	3.7e-05
*GPoiss*	$\hat{\lambda }=4.99$	$\hat{p}=0.70$	189.56	383.12	386.95	0.5	1.05e-07
*DGeo*	–	$\hat{p}=0.06$	192.53	387.06	388.97	0.5	1.02e-07
*DGE*_2_	$\hat{\alpha }=1.84$	$\hat{p}=0.92$	188.48	380.97	384.79	0.5	1.05e-07
*Poiss*	$\hat{\lambda }=16.80$	–	644.51	646.51	648.42	0.5	1.08e-07

**Table 11:** Parameters estimates, -log (L), AIC, BIC, (*K*–*S*) and P-value (p-v) of *DMPEII*, *DBuur*, *DGPoiss*, *DGeo*, ${DGE}_{2}$, and *DPoiss* distributions for (dataset2-G2).

Dist.	Parameters Estimates		-log(L)	AIC	BIC	*K*–*S*	p-v
*DMPEII*	$\hat{\alpha }=1.13$	–	**189.2**	**380.5**	**382.4**	**0.1**	**0.5287**
*DBurr*	$\hat{\alpha }=59.97$	$\hat{\theta }=0.99$	222.08	448.16	452.03	0.3	0.0002
*DGPoiss*	$\hat{\lambda }=4.6$	$\hat{p}=0.7$	189.6	383.3	387.1	0.5	8.8e-07
*DGeo*	–	$\hat{p}=0.06$	192.7	387.4	389.3	0.5	8.5e-07
*DGE*_2_	$\hat{\alpha }=1.8$	$\hat{p}=0.9$	189.9	381.99	385.9	0.5	8.8e-07
*DPoiss*	$\hat{\lambda }=15.6$	–	331.6	665.2	667.1	0.5	9.1e-07

Because the p-value is greater than the significance level of 0.05, it can be concluded that the data are consistent with a *DMPEII* model. As a result, evidence supports the claim that *DMPEII* accurately describes this dataset and outperforms other distributions that fit the proposed distribution.

**Fig 12:**
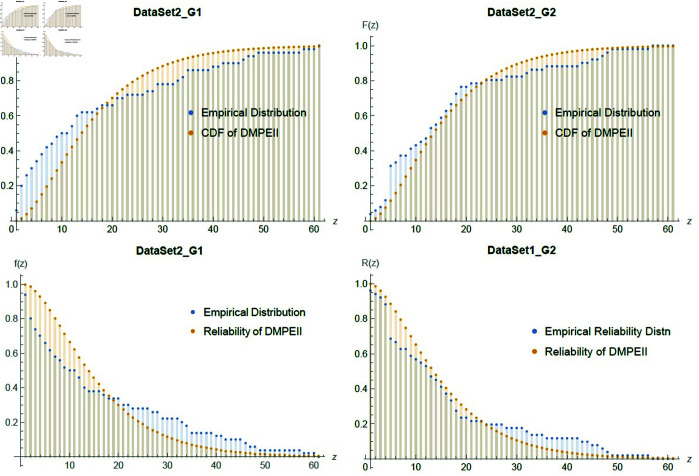
Empirical fitting graph between cdf & reliability and two groups patients of Dataset2 with *DMPEII* distribution.

Comparing the efficiency of these two transplant methods based on the time in which patients experience leukemia-free reliability, that is, the time they are disease-free after their transplants, is an important question in bone marrow transplantation. To answer this question, we can observe in the Table 12 and Fig 12, which compares the two groups of patients with different treatments by comparing the hazard function, the reliability function and the mean remision time tests of G2, is superior to G1 because it has a high p-value and a low mean remision time.

**Table 12:** Comparison between (Dataset2-G1) and (Dataset2-G2).

Parameters Estimates	G1	G2
$\hat{\alpha }$	1.134	1.138
${\hat{m}}_{1}=E(Z){|}_{\hat{\alpha }}$	18.95	18.48
${\hat{hr}}_{DMPEII}({\hat{m}}_{1};\hat{\alpha })$	0.08	0.08
${\hat{R}}_{DMPEII}({\hat{m}}_{1};\hat{\alpha })$	0.3909	0.3905
*p*–*value*	0.37	0.53

### 4.3 Dataset3

The third dataset (Dataset3) in Table 13, released by Lawless [13], indicates the lengths of remission in weeks for a group of 30 leukemia patients taking a specific kind of medicine.

**Table 13:** Remission times (in weeks) for 30 leukemia patients taking a specific type of therapy (dataset3).

1	1	2	2	2	2	2	2	4	4
4	4	5	5	5	6	6	7	7	8
9	9	10	12	13	18	18	20	40	40

**Fig 13:**
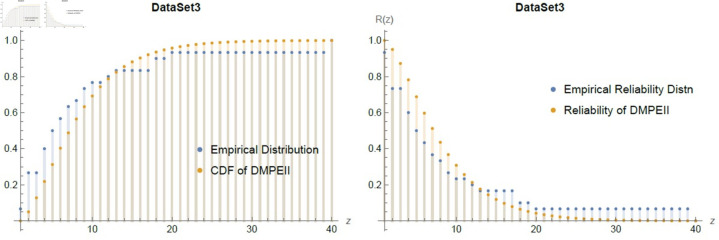
Empirical fitting graph of cdf & reliability between Dataset3 and *DMPEII* distribution.

Table 14 hows that the proposed distribution is the only one that fits the dataset3, with the lowest K-S value and the largest P-values when compared to the other distributions. Moreover, Fig 13, presented the goodness of fit between the dataset3 and *DMPEII* among the distribution and reliability functions.

**Table 14:** Parameters estimates, -log (L), AIC, BIC, (*K*–*S*) and P-value (p-v) of *DMPEII*, *DBuur*, *DGPoiss*, *DGeo*, ${DGE}_{2}$, and *DPoiss* distributions for (dataset3).

Dist.	Parameters Estimates		-log(L)	AIC	BIC	*K*–*S*	p-v
*DMPEII*	$\hat{\alpha }=1.25$	–	**93.05**	**188.09**	**189.5**	**0.15**	**0.497**
*DBurr*	$\hat{\alpha }=208.4$	$\hat{\theta }=0.99$	96.7	197.4	200.2	0.25	0.05
*DGPoiss*	$\hat{\lambda }=3.04$	$\hat{p}=0.6$	93.1	188.3	191.1	0.5	0.0005
*DGeo*	–	$\hat{p}=0.1$	94.07	190.1	191.5	0.5	0.0005
*DGE*_2_	$\hat{\alpha }=1.69$	$\hat{p}=0.85$	93.3	188.6	191.4	0.5	0.0005
*DPoiss*	$\hat{\lambda }=7.97$	–	141.1	284.3	285.7	0.5	0.0005

### 4.4 Dataset4

The fourth dataset (Dataset4) in Table 15, provided by Klein Moeschberger [14] describes the death times, expressed in weeks, of 30 tongue cancer patients.

**Table 15:** Death times (in weeks) of 30 patients with cancer of the tongue (dataset4).

1	2	3	4	4	4	4	4	4	4
5	6	7	7	8	10	12	15	16	18
20	20	27	27	27	28	30	30	32	35

**Fig 14:**
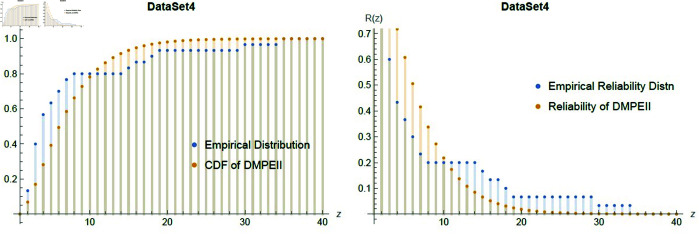
Empirical fitting graph of cdf & reliability between Dataset4 and *DMPEII* distribution.

Fig 14 shows a good fit between dataset3 and *DMPEIID* in terms of distribution and reliability functions. Furthermore, Table 16 indicates that the proposed distribution fits dataset 4 better than the competing models, with the lowest *K*–*S* and largest P-values compared to the other distributions.

**Table 16:** Parameters estimates, -log (L), AIC, BIC, (*K*–*S*) and P-value (p-v) of *DMPEII*, *DBuur*, *DGPoiss*, *DGeo*, ${DGE}_{2}$, and *DPoiss* distributions for (dataset4).

Dist.	Parameters Estimates		-log(L)	AIC	BIC	*K*–*S*	p-v
*DMPEII*	$\hat{\alpha }=1.15$	–	**108.09**	**219.2**	**220.6**	**0.2**	**0.3956**
*DBurr*	$\hat{\alpha }=36.19$	$\hat{\theta }=0.99$	124.43	252.9	255.7	0.4	0.001
*DGPoiss*	$\hat{\lambda }=4.06$	$\hat{p}=0.7$	108.26	220.5	223.3	0.5	5.6e-05
*DGeo*	–	$\hat{p}=0.07$	109.8	221.6	223.01	0.5	5.4e-05
*DGE*_2_	$\hat{\alpha }=1.6$	$\hat{p}=0.9$	108.16	220.3	223.1	0.5	5.5e-05
*DPoiss*	$\hat{\lambda }=13.8$	–	190.4	382.9	384.3	0.5	6.5e-05

## 5 Conclusion

This article uses a Poisson distribution mixture technique to build a discrete mixed Poisson-Erlang distribution of one parameter (*DMPED*) by mixing a continuous two-parameter Erlang distribution with the Poisson distribution. We calculate several key statistical functions for the proposed distribution, including reliability, hazard rate, reverse hazard rate, Mills ratio, and inverse distribution (quantile) functions. Numerous statistical characteristics of the proposed distribution are explored, notably measures of central tendency, dispersion, monotonicity, moments, probability and moment-generating functions, the estimation model parameter using the maximum likelihood technique, and a simulation study is provided. Furthermore, the proposed distribution is unimodal (obtains this mode) and has an advantageous impact on overly dispersed datasets, as measured by the Fano factor as the rate parameter grows.

Finally, we tested the proposed distribution on four different types of medical datasets to establish its efficacy. Dataset 1 shows the average number of doses required to cure 35 breast cancer patients, tailored to tumor recovery. Dataset 2 compares two groups of acute leukemia patients’ approaches with two different therapies undergoing bone marrow transplantation. The third and fourth datasets illustrate the likelihood of the tumor vanishing following therapy, the number of weeks that patients with tongue cancer were treated, and the duration between remision and death. (Eventually, we want to present a work in this statistical field of medicine).
